# Improvement in sequelae of chronic liver disease in patients with Alagille syndrome treated with maralixibat

**DOI:** 10.1002/jpr3.70262

**Published:** 2026-11-12

**Authors:** Tin Bo Nicholas Lam, Maria Paulina Ordonez, Amber Hidlreth

**Affiliations:** ^1^ Department of Pediatrics, Division of Gastroenterology, Hepatology, and Nutrition University of California San Diego School of Medicine La Jolla California USA

**Keywords:** cholestasis, ileal bile acid absorption transporter inhibitor, varices, xanthomas

## Abstract

Maralixibat (MRX) is an orally administered, small‐molecule ileal bile acid transporter (IBAT) inhibitor used in the treatment of cholestatic pruritus in conditions such as Alagille syndrome (ALGS). MRX's beneficial effects on cholestatic pruritus are well described, however its effects on patients’ clinical manifestations of hepatic disease are less well known. We report two cases of patients with ALGS treated with MRX who demonstrated stabilization of their esophageal varices and xanthomas, respectively. Our findings demonstrate the potential role of MRX in improving the extrahepatic manifestations of liver disease in patients with ALGS. Further studies are needed to understand the physiologic mechanisms driving these changes.

## Abstract (highlights)

**What is Known:**

- Alagille syndrome (ALGS) is a multisystem disorder with significant hepatobiliary disease burden.
- Maralixibat (MRX), an orally administered, small‐molecule ileal bile acid transporter (IBAT) inhibitor improves cholestatic pruritus and quality of life in individuals with ALGS.

**What is New:**

- MRX may improve dyslipidemia and xanthomas in those with ALGS.
- MRX may improve esophageal varices in individuals with ALGS.

## 1 INTRODUCTION

Maralixibat (MRX) is an orally administered, small‐molecule ileal bile acid transporter (IBAT) inhibitor used in the treatment of cholestatic pruritus in conditions such as Alagille syndrome (ALGS).^1^, ^2^ MRX's effect on reducing cholestatic pruritus has previously been documented.^3^ However, its effects on a patient's primary hepatic disease process and/or the clinical consequences of hepatic disease are less well known. We present two cases documenting stabilization of esophageal varices and improvement in xanthomas after MRX initiation in two patients with ALGS.

## 2 CASE 1

A 10‐year‐old male with ALGS (*JAG1* c.62_73del, p.Leu21_Leu24del), left pulmonary artery hypoplasia (mild gradient), left to right atrial shunting, portal hypertension (presenting at 2 years of age with splenomegaly, and thrombocytopenia), active cholestatic pruritus managed with rifampin and antihistamines as needed, was seen in routine outpatient follow‐up. On exam, splenomegaly was appreciated with spleen border palpable 7 cm below the left costal border in the midclavicular line. Laboratory tests showed a white blood cell count (WBC) 1.7 K/mcL, platelet count 66 K/mcL, alanine aminotransferase (ALT) 110 U/L, aspartate aminotransferase (AST) 103 U/L, bilirubin total (TB) 1.2 mg/dL, bilirubin direct (DB) 0.6 mg/dL, gamma‐glutamyl transferase (GGT) 567 unit/L, international normalized ratio (INR) 1.0, serum bile acids (sBA) 53 µmol/L and no evidence of fat soluble vitamin deficiency (Table 1a). He was previously treated with ursodiol, with reported worsening of pruritus on this agent. Thus, at this visit, he was transitioned off rifampin and onto MRX 190 mcg/kg orally, once daily starting days 1−7, then increased to 380 mcg/kg orally, once daily beginning day 8.

**Table 1a:** Case 1 laboratory tests pre‐ and post‐maralixibat initiation.

Laboratory value	Pre‐maralixibat	3‐months post‐maralixibat	1‐year post‐maralixibat
WBC K/mcL	1.7	1.6	2.1
Platelets, K/mcL	66	57	48
ALT, U/L	110	299	347
AST, U/L	103	183	224
TB, mg/dL	1.2	1.0	1.2
DB, mg/dL	0.6	0.4	0.2
GGT, U/L	567	601	626
INR	1.0	1.0	1.1
sBA µmol/L	53	12	18
Vitamin A (retinol/retinyl palmitate) mg/L	0.53/0.13	‐	0.59/<0.02
Vitamin D, ng/mL	64	‐	65
Vitamin E (alpha‐tocopherol/gamma‐tocopherol), mg/L	8.9/1.5	‐	9.4/1.2
HDL, mg/dL	65	‐	62
LDL, direct mg/dL	124	‐	95
Non‐HDL cholesterol (LDL + very LDL) mg/dL	146	‐	128
TG, mg/dL	112	‐	167

*Note*: Laboratory values (including select results from complete blood counts, comprehensive metabolic panels, coagulation studies, bile acids, fat soluble vitamin levels, and lipid panels) for case 1 prior to initiation of maralixibat, then 3 months after initiation and 1‐year after initiation of maralixibat.

Abbreviations: ALT, alanine aminotransferase; AST, aspartate aminotransferase; DB, bilirubin direct; GGT, gamma‐glytamyl transferase; HDL, High density lipoprotein; INR, international normalized ratio; LDL, Low‐density lipoprotein; sBA, serum bile acids; TB, bilirubin total; TG, Triglycerides; WBC, white blood cell count.

At follow‐up 3 months later, his pruritus had resolved and labs were notable for ALT 299 U/L, AST 183 U/L, sBA 12 µmol/L (Table 1a). Surveillance esophagogastroduodenoscopy (EGD) was performed demonstrating four columns of grade 1 varices in the distal esophagus (Figure 1A); no band ligation was required. He continued on MRX in the interim.

**Figure 1:**
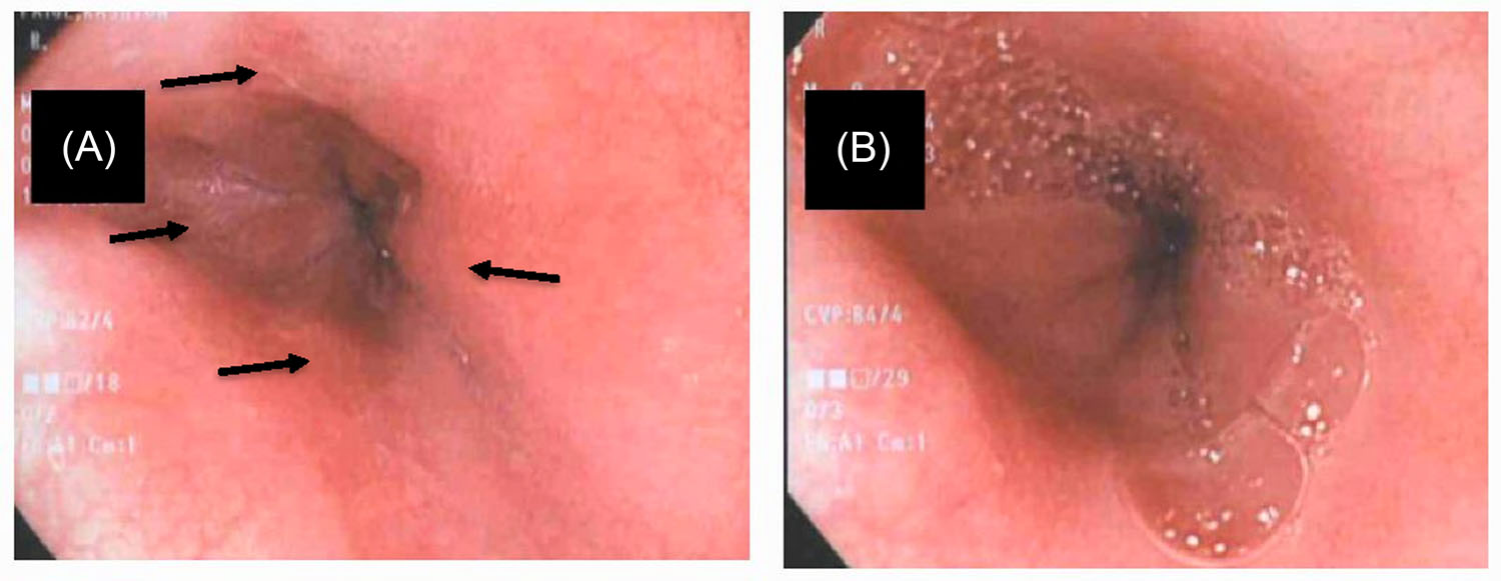
(A) EGD image of distal esophagus demonstrating four columns of grade 1 esophageal varices. (B) EGD image of distal esophagus 1 year later with resolution of varices. EGD, esophagoduodenoscopy.

At follow‐up 1 year later, the patient remained clinically stable with exam notable for a palpable spleen edge 9.5 cm below the left costal border in the midclavicular line. Labs were notable for platelets 48 K/mcL, ALT 347 U/L, AST 224 U/L, sBA 18 µmol/L; bilirubin, GGT, INR and fat soluble vitamin levels remained stable (Table 1a). A repeat surveillance EGD performed by the same endoscopist demonstrated visual improvement of esophageal varices (Figure 1B).

## 3 CASE 2

A 2‐year‐old female with ALGS (*JAG1*, c.1890_1893del, p.Ile630Metfs*112), spontaneously closed ventricular septal defect, mild branch pulmonary artery stenosis, vascular ring, absent bilateral internal carotid arteries, hypertension, splenomegaly, cholestasis, fat‐soluble vitamin deficiency, and pruritus (managed with ursodiol and rifampin) was seen in outpatient follow‐up. On exam, she was noted to have widespread jaundice, scleral icterus, mild distress from itching, and a cluster of circular papules in her right inguinal region and chest consistent with xanthomas (Figure 2A). Lab testing was notable for platelets 231 K/mcL, ALT 253 U/L, AST 240 U/L, TB 25.7 mg/dL, DB 19 mg/dL, INR 1.02, GGT 1028 U/L, total sBA 349.5 µmol/L, triglycerides (TG) 382 mg/dL, cholesterol 1041 mg/dL, high density lipoprotein (HDL) 29 mg/dL, low density lipoprotein (LDL) >599 mg/dL, normal vitamin A 61 mcg/dL, low vitamin D 9.1 ng/mL, and normal vitamin E (alpha‐tocopherol) 3.6 mg/L (Table 1b).

**Figure 2:**
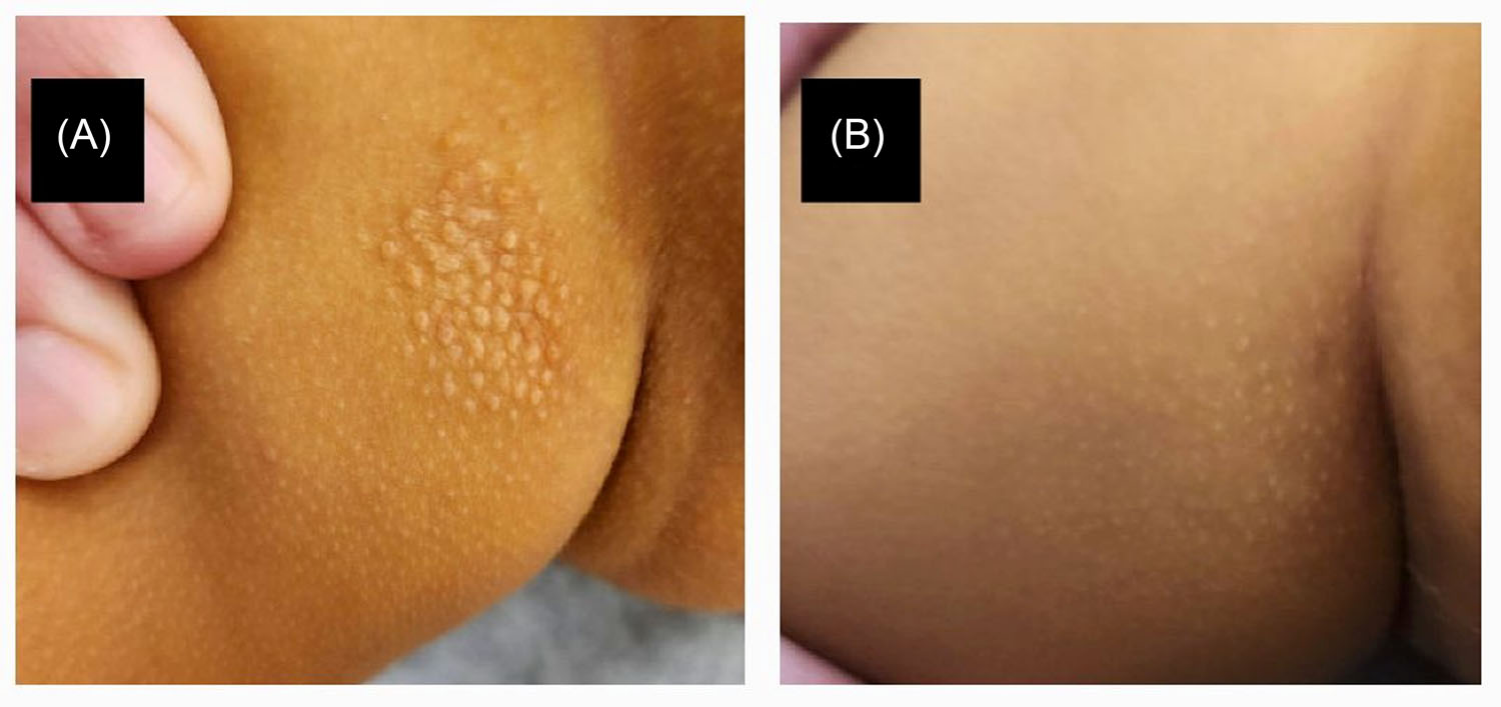
(A) Image of groin/inguinal region demonstrating cluster of xanthomas. (B) Image of groin/inguinal region 1 year later.

**Table 1b:** Case 2 laboratory tests pre‐ and post‐maralixibat initiation.

Laboratory value	Pre‐maralixibat	1‐year post‐maralixibat
WBC, K/mcL	6.6	5.8
Platelets, K/mcL	231	110
ALT, U/L	253	462
AST, U/L	240	708
TB, mg/dL	25.7	16.4
DB, mg/dL	19	13
GGT, U/L	1028	376
INR	1.02	1.06
BA µmol/L	349.5	‐
Vitamin A (retinol/retinyl palmitate) mg/L	61	24
Vitamin D, ng/mL	9.1	13.9
Vitamin E (alpha‐tocopherol/gamma‐tocopherol), mg/L	3.6/<1	1.9/<1
Cholesterol total, mg/dL	1041	303
HDL, mg/dL	29	15
LDL, direct mg/dL	>599	237
TG, mg/dL	382	252

*Note*: Laboratory values (including select results from complete blood counts, comprehensive metabolic panels, coagulation studies, bile acids, fat soluble vitamin levels, and lipid panels) for case 2 prior to initiation of maralixibat and 1‐year after initiation of maralixibat.

Abbreviations: ALT, alanine aminotransferase; AST, aspartate aminotransferase; BA, bile acids; DB, bilirubin direct; GGT, gamma‐glytamyl transferase; HDL, High density lipoprotein; INR, international normalized ratio; LDL, Low‐density lipoprotein; TB, bilirubin total; TG, Triglycerides; WBC, white blood cell count.

She was initiated on MRX 190 mcg/kg orally, once daily starting days 1–7, then increased to 380 mcg/kg orally, once daily starting day 8 and thereafter.

At follow‐up 1 year later, her pruritus was significantly improved, with intermittent flares. Examination demonstrated near resolution of the xanthomas (Figure 2B). Labs were notable for ALT 462 U/L, AST 708 U/L, TB 16.4 mg/dL, DB 13 mg/dL, INR 1.06, GGT 376 U/L, TG 252 mg/dL, cholesterol 303 mg/dL, LDL cholesterol 237 mg/dL, normal vitamin A level 24 mcg/dL, improved vitamin D level 13.9 ng/mL, and decreased vit E (alpha‐tocopherol) 1.9 mg/L (Table 1b).

## 4 DISCUSSION

Both patients treated with MRX demonstrated lower serum bile acid levels and improved pruritus at 1 year of therapy. These findings echo those seen in the ICONIC trial, which details MRX's effect on reducing sBA and cholestatic pruritus in children with ALGS.^3^ This improvement in pruritus has not only been associated with improved QOL, but also improved event‐free survival and transplant‐free survival rates.^4^, ^5^ Our patients’ experiences highlight the utility of MRX in the management of refractory cholestatic pruritus.

Both patients’ ALT levels increased after starting MRX, a finding noted in previous literature where individuals with ALGS on MRX developed elevations in ALT, AST, and/or TB/DB over time. While ALT fluctuations over time may be a result of the natural course of those with ALGS, drug‐induced liver injury (DILI) has been proposed as an alternative etiology, and has been reported in individuals with PFIC taking MRX.^6^, ^7^, ^8^ Neither patient demonstrated worsened coagulation studies, bilirubin nor albumin levels. Additionally, neither patient reported diarrhea, abdominal pain, nausea/vomiting, or fractures. Neither of our patients required dose reduction nor cessation of therapy. The elevations in our patients’ ALT/AST over time without other hepatic function disturbances likely reflect the natural progression of their liver disease.

Portal hypertension secondary to cirrhosis is a common reason for liver transplantation in children with ALGS. Despite persistent splenomegaly and thrombocytopenia, our first case displayed endoscopic stabilization of grade 1 varices after starting MRX, without concurrent anti‐hypertensive pharmacotherapy or changes in cardiac function. Hepatic venous pressures were not performed pre‐ or post‐initiation of MRX. To our knowledge, no studies have evaluated the effect of MRX on portal hypertension in ALGS.

Our second patient demonstrated improvement in their xanthomas and dyslipidemia after initiating MRX. The effect of MRX on xanthomas has been detailed by Gonzalez et al. in the ICONIC study, where 14 individuals with xanthomas at study enrollment were noted to have improvement in clinician xanthoma scores at 48 and 204 weeks follow‐up.^3^ Dyslipidemia and xanthoma formation are common in children with ALGS,^6^ and are associated with cholestasis, causing abnormal lipid metabolism, low lecithin‐cholesterol‐acyl‐transferase activity, an accumulation of unesterified cholesterol and the formation of lipoprotein‐X (Lp‐X).^9^ Due to similarities in density, standard lab assays cannot distinguish between Lp‐X and LDL, leading to elevated LDL levels in routine lipid profiles of patients with ALGS. Because Lp‐X is catabolized independently from normal cholesterol metabolic pathways, the elevated LDL seen in ALGS patients does not respond to standard lipid‐lowering therapies (i.e., statins).^10^ Despite this, Lp‐X elevations in ALGS are not typically associated with increased risk of cardiovascular disease nor atherosclerosis.^9^, ^11^ Our patient's improvements in TG, cholesterol and LDL are findings not previously described, and may be a result of decreased bile acid circulation limiting intestinal fat absorption and the development of Lp‐X. MRX may worsen existing fat‐soluble vitamin deficiencies in children with ALGS on MRX, however its ability to cause deficiencies is not described. Thus, it is unclear whether the new vitamin E deficiency noted in our second patient was associated with starting MRX. Further studies are needed to examine MRX's role in lipid metabolism in ALGS.

## 5 CONCLUSION

Our findings demonstrate the potential role of MRX in improving the manifestations of chronic liver disease, including esophageal varices, dyslipidemia and xanthomas, in patients with ALGS. Further studies are needed to understand the physiologic mechanisms driving these changes.

## CONFLICT OF INTEREST STATEMENT

Dr. Amber Hildreth currently consults and speaks for Ipsen and Mirium pharmaceuticals. The remaining authors declare no conflicts of interest.

## ETHICS STATEMENT

Informed consent was obtained from the respective parent/legal guardian of each individual.
